# Obese Patient with Gastric Diverticulum Undergoing Laparoscopic Sleeve Gastrectomy Guided by Preoperative Endoscopic Measurement: A Case Report and Literature Review

**DOI:** 10.70352/scrj.cr.25-0141

**Published:** 2025-06-10

**Authors:** Kensuke Hirosuna, Hajime Kashima, Ryohei Shoji, Yuki Matsumi, Yoshihiko Kakiuchi, Satoru Kikuchi, Shinji Kuroda, Fuminori Teraishi, Shunsuke Kagawa, Toshiyoshi Fujiwara

**Affiliations:** 1 Center for Graduate Medical Education, Okayama University Hospital, Okayama, Okayama, Japan; 2 Department of Gastroenterological Surgery, Okayama University Graduate School of Medicine, Dentistry, and Pharmaceutical Sciences, Okayama, Okayama, Japan

**Keywords:** obese patient, gastric diverticulum, sleeve gastrectomy, metabolic surgery, bariatric surgery, endoscopic measurement

## Abstract

**INTRODUCTION:**

Gastric diverticulum is a rare condition, often asymptomatic and incidentally detected. Laparoscopic sleeve gastrectomy (LSG) is a widely performed bariatric procedure, but a gastric diverticulum complicates surgical planning. In this case, careful preoperative assessment allowed safe execution of LSG despite the diverticulum’s proximity to the esophagogastric junction.

**CASE PRESENTATION:**

A 45-year-old woman (BMI: 46.8 kg/m^2^) with hypertension, dyslipidemia, and glucose intolerance was referred for bariatric surgery after unsuccessful weight loss with conservative management. Preoperative endoscopy revealed an 18 × 14 mm gastric diverticulum on the posterior wall of the gastric fundus, 40 mm from the esophagogastric junction. LSG was performed using a surgical stapler, ensuring complete diverticulum resection while preserving gastric tube integrity. The surgery was uneventful, with minimal blood loss and a duration of 2 hours and 52 minutes. The patient had an uneventful postoperative course and was discharged on day 9. Her BMI decreased to 39.3 kg/m^2^ at the 1-year follow-up, with improved metabolic parameters.

**CONCLUSIONS:**

This case highlights the importance of thorough preoperative evaluation when performing LSG in patients with gastric diverticulum. Accurate endoscopic measurement of the diverticulum’s location aids in determining the optimal resection line, ensuring surgical safety and efficacy. Surgeons should remain vigilant when encountering such anatomical variations to optimize outcomes in bariatric surgery.

## INTRODUCTION

Gastric diverticulum is a rare disease among gastrointestinal diverticula, with a reported prevalence of 0.01%–0.11%.^1)^ Laparoscopic sleeve gastrectomy is one of the weight loss methods for patients with severe obesity,^2)^ and its use has been increasing in recent years.^3–5)^ Resection of greater curvature is an essential procedure in sleeve gastrectomy and requires careful determination of the line of separation. In this case, the distance between the esophagogastric junction and the diverticulum was closer, making it more challenging to determine the dissection line than usual. We performed a laparoscopic sleeve gastrectomy on an obese patient with a gastric diverticulum. Measuring the distance with a device during preoperative endoscopy is key to determining the appropriate incision line for obese patients complicated with gastric diverticulum.

## CASE PRESENTATION

The patient is a 45-year-old woman. Physical examination at the initial consultation as follows: height: 156.6 cm; weight: 114.8 kg; BMI: 46.8 kg/m^2^.

### Current medical history

She was followed by her family doctor for hypertension and dyslipidemia. She was under the care of a dietitian but was referred to our clinic because she was not losing weight. After 6 months of medical treatment, she lost 101.3 kg and a BMI of 41.3 kg/m^2^, but further weight loss was difficult, and the patient was referred for surgery to improve weight loss and metabolism.

### History

Dyslipidemia, hypertension, and glucose intolerance (borderline type).

### Blood biochemical test findings

HbA1c: 5.8%; FBS: 98 mg/dL; total cholesterol: 143 mg/dL; LDL-C: 73 mg/dL; HDL-C: 55 mg/dL; and TG: 72 mg/dL.

### Upper gastrointestinal endoscopy

**Fig. 1:**
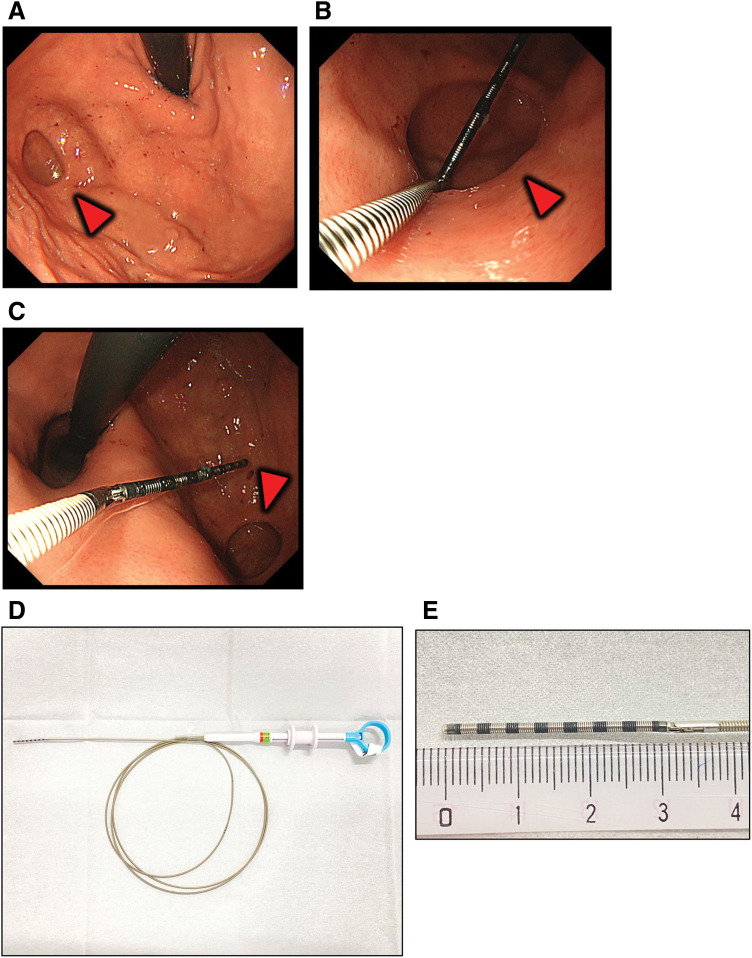
Preoperative upper gastrointestinal endoscopic images. The red triangle points to the diverticulum. (**A**) Diverticulum on the posterior wall of the fundus of the stomach. (**B**) The distance from the esophagogastric junction to the diverticulum is measured with a probe. (**C**) Measurement of the diverticulum with a probe revealed a short diameter of about 14 mm. (**D**) Device used to measure distance during endoscopy (M2-4K; Olympus, Tokyo, Japan). (**E**) High magnification; 1 black line or silver line indicates 2 mm.

An 18 × 14 mm gastric diverticulum was detected on the dorsal surface of the gastric fundus (**Fig. 1A** and **1B**). When measured with a probe, the distance between the esophagogastric junction and the diverticulum was 40 mm (**Fig. 1C**). An endoscopic bendable measuring probe (M2-4K; Olympus, Tokyo, Japan) was used for endoscopic length measurements (**Fig. 1D** and **1E**).

### CT (**Fig. 2**)

A diverticular lesion protruding outside the wall was observed in the gastric fundus.[Fig F2]

**Fig. 2 F2:**
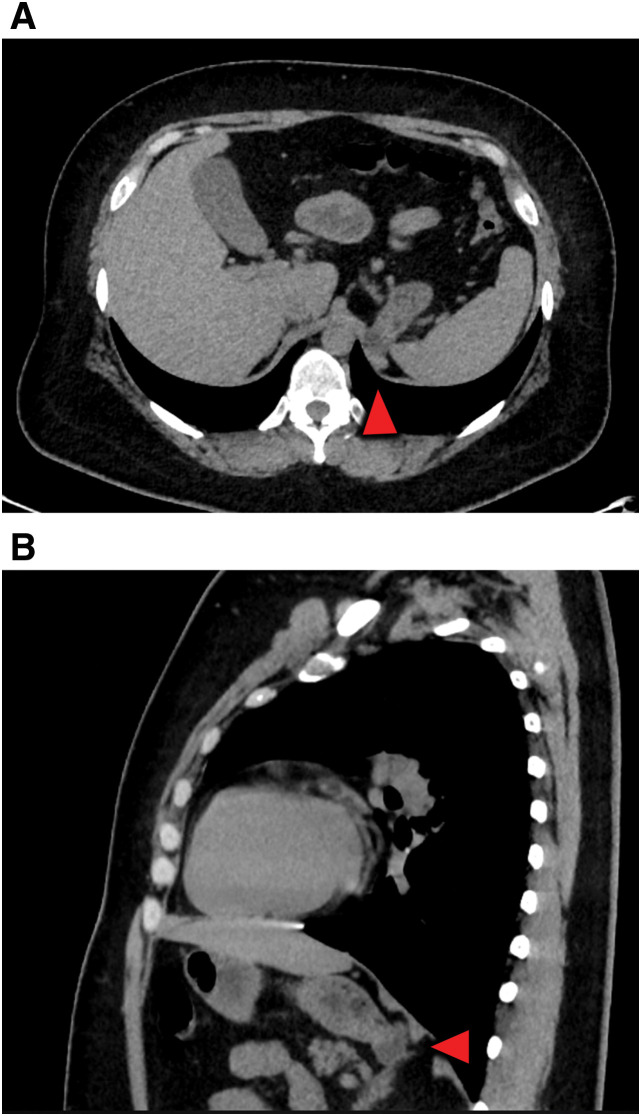
Preoperative plain computed tomography images. A diverticulum protruding dorsally at the fundus of the stomach is observed. The red triangle points to the diverticulum. (**A**) Axial and (**B**) sagittal.

Preoperative endoscopy showed 40 mm to the diverticulum, which was considered to be included in the resected gastric side, and laparoscopic sleeve gastrectomy was planned as usual.

### Surgical findings (**Fig. 3**)

Surgery started with 4 ports of 15, 12, 12, and 5 mm. After dissecting the gastric mesentery, the dorsal fundus was dissected along the gastric wall to identify the diverticulum. As preoperatively estimated, the diverticulum was well distanced from the esophagogastric junction. Dissection was initiated from the gastric antrum with a surgical stapler. The position of the stapler was adjusted so that the diverticulum could be resected while maintaining an appropriate gastric tube diameter. Gastrectomy on the greater curvature side was completed, the resected stomach was removed from the body, and the operation was completed without trouble. Blood loss was 50 mL, and operating time was 2 hours and 52 minutes. A postoperative specimen revealed a diverticulum protruding on the posterior wall of the fundus of the stomach. There were no malignant or inflammatory findings, and pathologically, the entire gastric wall was confirmed and diagnosed as a true diverticulum [Fig F3](**Fig. 4**). Fluid intake started the day after surgery, and food intake started on the third postoperative day. The patient had no postoperative complications and was discharged from the hospital 9 days after surgery. Three months after surgery, an upper gastrointestinal endoscopy revealed no deformation or stricture of the gastrointestinal tract, and the suture line was clean (**Fig. 5**). One year after surgery, her weight was 96.4 kg, and her BMI was 39.3 kg/m^2^, indicating a significant weight loss effect. Blood chemistries showed HbA1c: 5.8%; fasting blood glucose: 108 mg/dL; total cholesterol: 209 mg/dL; LDL-C: 117 mg/dL; HDL-C: 80 mg/dL; and TG: 86 mg/dL. All values were within the standard range, and the patient’s condition was stable.

**Fig. 3 F3:**
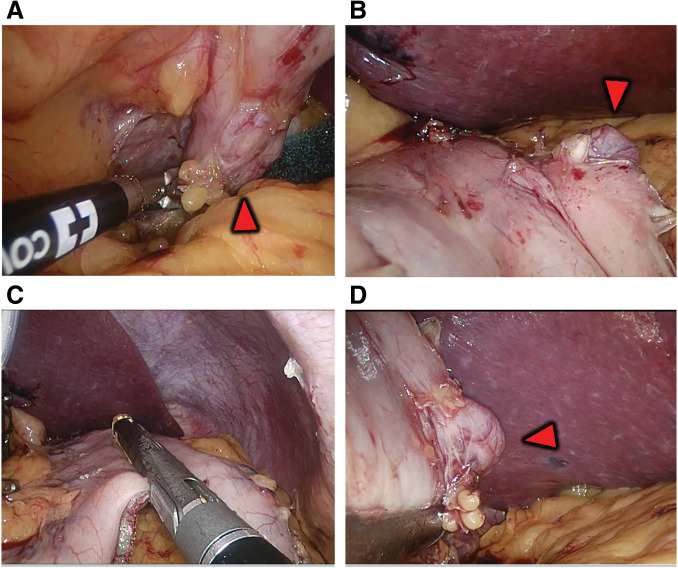
Intraoperative findings. The red triangle points to the diverticulum. (**A**) The mesentery of the dorsal gastric wall is dissected. (**B**) The gastric wall is grasped as if to turn it over to check the dorsal gastric diverticulum. (**C**) Suture dissection of the stomach is proceeding with a surgical stapler. (**D**) The dissection line is visually determined on the dorsal side and instrumented dissection is perfomed.

**Fig. 4:**
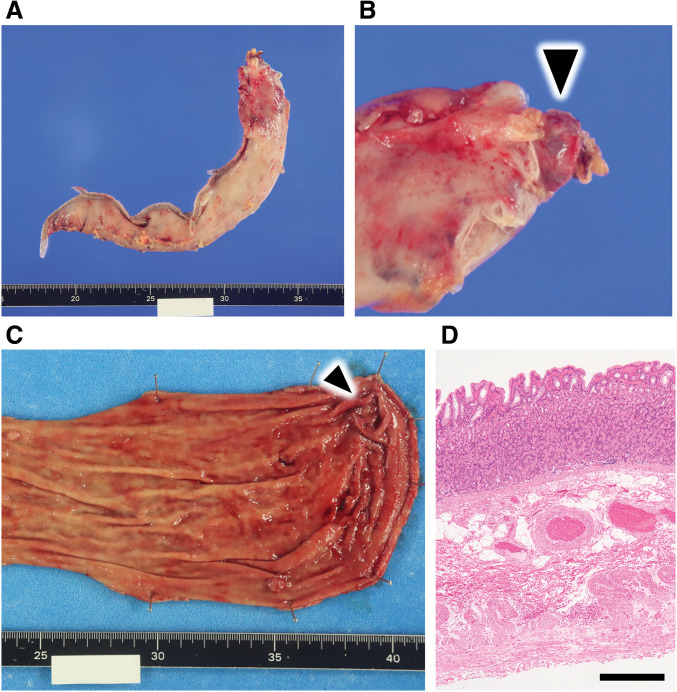
Pictures of surgical specimens. (**A**) Resected stomach before opening. (**B**) High magnification. The black triangle indicates the diverticulum outside the stomach. (**C**) The diverticulum is identified from the mucosal side of the stomach. The black triangle indicates the diverticulum. (**D**) Hematoxylin–eosin staining of a histopathology specimen of the diverticular site. The scale bar is 500 μm.

**Fig. 5:**
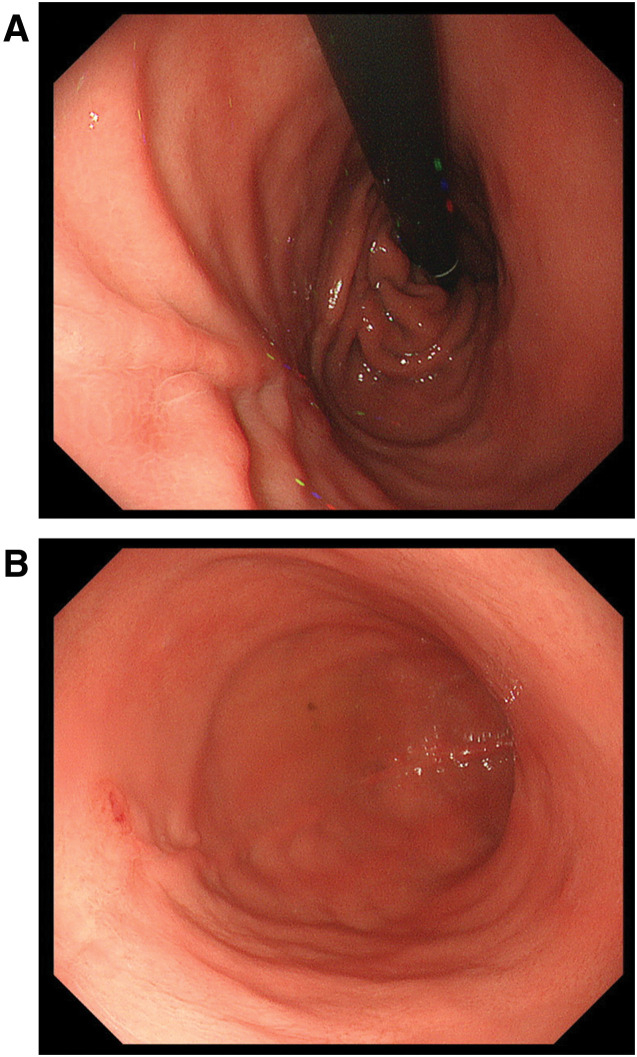
Postoperative upper gastrointestinal endoscopic images. The incision line is clean, and there is no deformity or stenosis. (**A**) View looking up the esophagus from the stomach side. (**B**) View looking down on the stomach.

## DISCUSSION

A gastric diverticulum is a rare disease, but our patient had an even rarer complication of a gastric diverticulum in an obese patient scheduled for sleeve gastrectomy. Careful preoperative upper gastrointestinal endoscopy to accurately measure the distance between the diverticulum and the esophagogastric junction, as well as the diameter of the diverticulum, was very helpful in performing a safe and reliable sleeve gastrectomy.

**Fig. 6:**
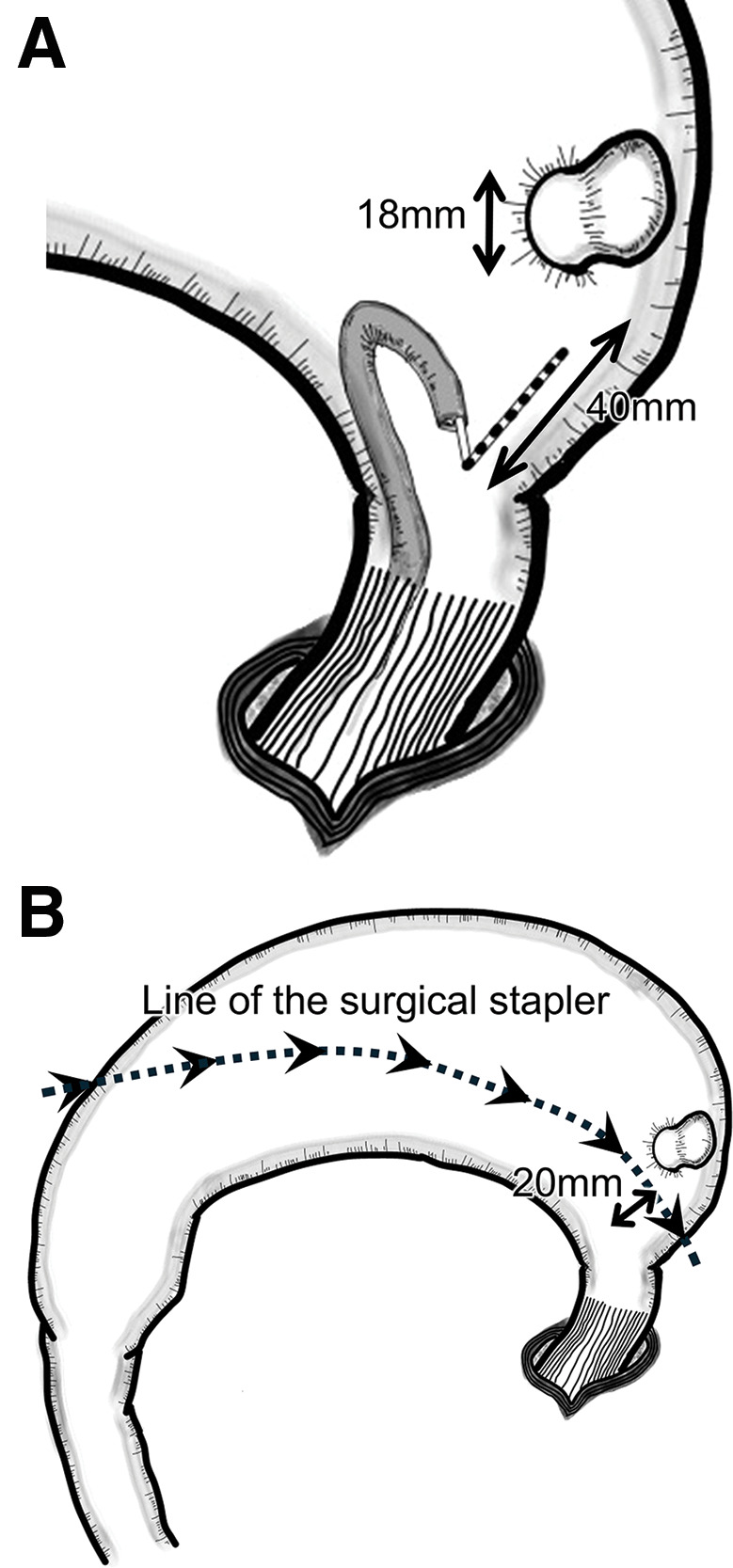
Schematic of the intraoperative endoscope and suturing by surgical stapler. The stomach is flipped from the greater curvature side. (**A**) The distance is measured endoscopically with a probe to confirm that suture separation can be done safely. (**B**) The diverticulum is resected and included on the resected gastric side.

Gastric diverticulum occurs almost equally in men and women, with an average age of 49.2 years (range 18–80). They are usually asymptomatic and are often discovered incidentally on examination.^6)^ Prevalence estimates range from 0.04% (165/380000) on upper gastrointestinal contrast radiography to 0.01%–0.11% on upper gastrointestinal endoscopy and 0.02% (6/29900) on autopsy examination.^1,7–9)^ If there are no symptoms, the patient is generally followed up. When accompanied by symptoms, the most common symptom is abdominal pain (48.2%),^6)^ which may be accompanied by nausea and vomiting, and medical treatment, including medication, may be performed. Serious complications include gastrointestinal bleeding and peritonitis due to perforation, which requires emergency surgery.^1,7)^ In this case, there were no clinical symptoms due to gastric diverticulum, which was first noted on a preoperative CT scan and upper gastrointestinal endoscopy. The mean maximum diameter of the gastric diverticulum is 3.97 cm (range 0.5–9).^6)^ It can occur in various sites, but the posterior wall of the gastric fundus is the predominant site in about 75% of cases. Gastric diverticula tend to form in this region because of the possibility of congenital defects in the gastric wall musculature or the presence of arterial perforation that contributes to the weakening of the gastric wall; pathologically, true diverticula are common.^1)^ True diverticula are those in which the entire gastric wall remains intact and are distinct from pseudodiverticula, in which the muscular layer is absent. When diverticula form near the pylorus or in areas other than the fundus of the stomach, such as the vestibule, they are pseudodiverticula since the internal pressure is often elevated due to obstruction of passage caused by pyloric stenosis.^6)^ In our case, the lesion was located on the posterior wall of the fundus, the predilection site. A histopathological specimen of the diverticulum area revealed all layers of the stomach wall, including the muscular layer, and a true diverticulum was diagnosed. This result was consistent with previous reports that diverticula occurring in the stomach’s fundus near the eruptive canal fundus are often true diverticula.^1,6)^ Gastrectomy on the greater curvature side is a surgical maneuver that requires great care, even when performing a normal sleeve gastrectomy. In this case, a diverticulum and its proximity to the esophagogastric junction necessitated additional care when dissecting the area around the fundus. The distance from the esophagogastric junction to the diverticulum was measured with a probe measure during the preoperative upper gastrointestinal endoscopy and was confirmed to be at least 40 mm. The diameter of the base of the diverticulum was also measured to be 18 mm, so we made a preoperative judgment that a safe sleeve gastrectomy, including the diverticulum, would be feasible (**Fig. 6A**). As a result, we created a gastric tube with an appropriate diameter without intraoperative troubles. In this report, preoperative fluoroscopy was not performed. Like upper gastrointestinal endoscopy, fluoroscopy is a beneficial examination to determine the diverticulum’s location, size, and shape. A more comprehensive evaluation may have been possible by combining upper gastrointestinal endoscopy and fluoroscopy, and a preoperative examination combining the two should be considered in the future.

The usual distance from the angle of His for a sleeve gastrectomy in our facility is 15–20 mm. In this case, the resection could be done in about 20 mm (**Fig. 6B**). A literature search for reports of sleeve gastrectomy for cases complicated by gastric diverticulum revealed 4 articles and 4 case reports^10–13)^ (**Table 1**). Three of the 5 cases were identified preoperatively, and 2 gastric diverticula were identified intraoperatively. Two cases in which a gastric diverticulum was identified intraoperatively were associated with intra-abdominal contamination from tissue damage. Fortunately, this did not lead to severe postoperative complications, but the report suggests the importance of preoperative examination. The decision regarding the line of dissection is one of the most critical decisions. However, it is challenging to determine a treatment strategy when the diverticulum is close to the esophagogastric junction. The diverticular tissue itself is more fragile than the normal gastric wall, so the resection line should not pass through the site of the diverticulum. Should the diverticulum be left on the residual gastric side? There is no unified opinion on handling cases where resection is not feasible, and further case studies are needed. However, theoretically, the diverticulum remaining in a smaller residual stomach is subject to high internal pressure, which increases the risk of diverticular enlargement and perforation. In one case, a diverticulum remained in the reduced stomach volume after gastric resection, although not after sleeve gastrectomy, and the symptoms became apparent, requiring surgical intervention.^10)^ Generally, good postoperative results have been obtained by resecting gastric diverticular lesions with a surgical stapler,^8,14)^ and it is desirable to leave the diverticulum in the residual stomach and resect only the diverticular site separately with a surgical stapler. Although not a gastric diverticulum, there have been some reports of obese patients with gastric submucosal tumors or GIST whose lesions are located close to the esophagogastric junction. In the case of submucosal tumors, the distance from the esophagogastric junction also makes it complicated to decide whether to include the lesion in the resected stomach or leave it in the residual stomach, and sleeve gastrectomy is used for GIST lesions that are located far enough from the EGJ to include the GIST in the resected stomach.^15)^ In cases where the lesion was located on the lesser curvature, a transgastric resection was performed, followed by a sleeve gastrectomy.^16)^ However, in the case of a diverticulum protruding outside the wall, transgastric resection is technically ineffective, and it is more realistic to resect only the lesion site we have described above. Care should be taken when performing the procedure in which the lesion remains in the remnant gastric tube, because if the incision suture line is too close to the greater curvature, the postoperative gastric tube may become too large, compromising the effect of weight loss and metabolic improvement.^17)^

**Table 1:** Review of sleeve gastrectomy for obesity with gastric diverticulum

Author	Year	Age	Sex	BMI at operation (kg/m^2^)	Size of diverticulum (mm)	Distance from the angle of His (or EGJ) (mm)	Additional notes
Surve et al.^10)^	2016	NA*	Female	37	30	10	Diverticulum after gastric imbrication
Castelli et al.^11)^	2019	47	Female	43	NA	NA	Diverticulum was perforated during dissection
		38	Female	39	NA	NA	
Smith et al.^12)^	2019	49	Female	42	30	NA	Diverticulum was ruptured during dissection
Wylie et al.^13)^	2020	34	Female	46	24	30	
Our case	2025	45	Female	41.3	18	40	

*Described as “middle-aged.”

BMI, body mass index; EGJ, esophagogastric junction; NA, not available.

## CONCLUSIONS

We experienced a case of laparoscopic sleeve gastrectomy in a highly obese patient with a gastric diverticulum, in which the diverticulum was included in the resected stomach, and the procedure was performed safely. The decision regarding the line of resection is crucial even in a conventional sleeve gastrectomy, but the difficulty is even more significant in cases with diverticulum complications. However, a careful preoperative upper gastrointestinal endoscopy, including measurement of the distance from the esophagogastric junction and the distance to the diverticulum, allows for safe sleeve gastrectomy even in cases of complicated gastric diverticula.

## ACKNOWLEDGMENTS

We would like to express our sincere gratitude to Dr. Takehiro Tanaka from the Department of Pathology, Okayama University Hospital (Okayama, Okayama, Japan) for performing the histological evaluations.

## DECLARATIONS

### Funding

None.

### Authors’ contributions

Kensuke Hirosuna (KH) drafted the original manuscript.

KH, Hajime Kashima (HK), Ryohei Shoji, Yuki Matsumi, Yoshihiko Kakiuchi, Satoru Kikuchi, Shinji Kuroda, Fuminori Teraishi, and Shunsuke Kagawa served as physicians for the patient.

KH and HK collected the data for the presented case.

HK edited the paper to produce the final version.

Toshiyoshi Fujiwara supervised the study.

All authors reviewed the manuscript and approved the final version.

### Availability of data and materials

The data supporting this study's findings are available from the corresponding author upon reasonable request.

### Ethics approval and consent to participate

According to Okayama University Hospital's guidelines, ethical approval was not required for this case report, as it is a single case study without experimental intervention. The principles of the Declaration of Helsinki were followed in preparing this report.

### Consent for publication

Informed consent was obtained from the patient for publication of this case report and accompanying images.

### Competing interests

The authors declare that they have no competing interests.

## References

[1] Gockel I Thomschke D Lorenz D. Gastrointestinal: gastric diverticula. J Gastroenterol Hepatol 2004; 19: 227. 14731136 10.1111/j.1440-1746.2004.3339b.x

[2] McCarty TR Jirapinyo P Thompson CC. Effect of sleeve gastrectomy on ghrelin, GLP-1, PYY, and GIP gut hormones: a systematic review and meta-analysis. Ann Surg 2020; 272: 72–80. 31592891 10.1097/SLA.0000000000003614

[3] Alalwan AA Friedman J Park H US national trends in bariatric surgery: a decade of study. Surgery 2021; 170: 13–7. 33714616 10.1016/j.surg.2021.02.002

[4] Angrisani L Santonicola A Iovino P Bariatric surgery survey 2018: similarities and disparities among the 5 IFSO chapters. Obes Surg 2021; 31: 1937–48. 33432483 10.1007/s11695-020-05207-7 PMC7800839

[5] Ohta M Kasama K Sasaki A Current status of laparoscopic bariatric/metabolic surgery in Japan: the sixth nationwide survey by the Japan consortium of obesity and metabolic surgery. Asian J Endosc Surg 2021; 14: 170–7. 32696619 10.1111/ases.12836 PMC8048478

[6] Shah J Patel K Sunkara T Gastric diverticulum: a comprehensive review. Inflamm Intest Dis 2019; 3: 161–6. 31111031 10.1159/000495463 PMC6501548

[7] Rodeberg DA Zaheer S Moir CR Gastric diverticulum: a series of four pediatric patients. J Pediatr Gastroenterol Nutr 2002; 34: 564–7. 12050587 10.1097/00005176-200205000-00019

[8] Schiller AH Roggendorf B Delker-Wegener S Laparoscopic resection of gastric diverticula: two case reports. Zentralbl Chir 2007; 132: 251–5. (in German) 17610199 10.1055/s-2007-960753

[9] Morris PD Allaway MGR Mwagiru DK Gastric diverticulum: a contemporary review and update in management. ANZ J Surg 2023; 93: 2828–32. 37743578 10.1111/ans.18707

[10] Surve A Zaveri H Cottam D. Symptomatic gastric diverticulum after gastric imbrication with conversion to sleeve gastrectomy. Surg Obes Relat Dis 2016; 12: 439–40. 26575354 10.1016/j.soard.2015.09.012

[11] Castelli AA Lutfi RE. Strategies for diagnosing and managing gastric diverticulum in sleeve gastrectomy. Surg Obes Relat Dis 2019; 15: 126–7. 30522869 10.1016/j.soard.2018.10.030

[12] Smith CR Whipple OC. Incidental gastric fundus diverticulum during laparoscopic sleeve gastrectomy. Am Surg 2019; 85: e12–3. 30760360

[13] Wylie LE Villalvazo Y Jensen C. A strong approach to a weak gastric wall in bariatric surgery: concurrent diverticulectomy and sleeve gastrectomy. Cureus 2020; 12: e6545. 32042521 10.7759/cureus.6545 PMC6996466

[14] Kim SH Lee SW Choi WJ Laparoscopic resection of gastric diverticulum. J Laparoendosc Adv Surg Tech A 1999; 9: 87–91. 10194699 10.1089/lap.1999.9.87

[15] Hashimoto K Sakaguchi Y Nambara S Laparoscopic sleeve gastrectomy performed in a morbidly obese patient with gastrointestinal stromal tumor: a case report and literature review. Surg Case Rep 2020; 6: 208. 32785860 10.1186/s40792-020-00976-w PMC7423818

[16] Çaynak M Özcan B. Laparoscopic transgastric resection of a gastrointestinal stromal tumor and concomitant sleeve gastrectomy: a case report. Obes Surg 2020; 30: 1596–9. 32060851 10.1007/s11695-020-04472-w

[17] Şahin K Güler SA Şimşek T The effect of residual gastric volume on body mass index, excess weight loss rate and metabolic response after sleeve gastrectomy. Chirurgia (Bucur) 2023; 118: 380–90. 37698000 10.21614/chirurgia.2023.v.118.i.4.p.380

